# Inner Ear Gene Transfection in Neonatal Mice Using Adeno-Associated Viral Vector: A Comparison of Two Approaches

**DOI:** 10.1371/journal.pone.0043218

**Published:** 2012-08-17

**Authors:** Li Xia, Shankai Yin, Jian Wang

**Affiliations:** 1 Department of Otolaryngology, Affiliated Sixth People's Hospital of Shanghai Jiao Tong University, Otolaryngology Institute of Shanghai Jiao Tong University, Shanghai, China; 2 School of Human Communication Disorder, Dalhousie University, Halifax, Nova Scotia, Canada; Innsbruck Medical University, Austria

## Abstract

Local gene transfection is a promising technique for the prevention and/or correction of inner ear diseases, particularly those resulting from genetic defects. Adeno-associated virus (AAV) is an ideal viral vector for inner ear gene transfection because of its safety, stability, long-lasting expression, and its high tropism for many different cell types. Recently, a new generation of AAV vectors with a tyrosine mutation (mut-AAV) has demonstrated significant improvement in transfection efficiency. A method for inner ear gene transfection via the intact round window membrane (RWM) has been developed in our laboratory. This method has not been tested in neonatal mice, an important species for the study of inherited hearing loss. Following a preliminary study to optimize the experimental protocol in order to reduce mortality, the present study investigated inner ear gene transfection in mice at postnatal day 7. We compared transfection efficiency, the safety of the scala tympani injection via RWM puncture, and the trans-RWM diffusion following partial digestion with an enzyme technique. The results revealed that approximately 47% of inner hair cells (IHCs) and 17% of outer hair cells (OHCs) were transfected via the trans-RWM approach. Transfection efficiency via RWM puncture (58% and 19% for IHCs and OHCs, respectively) was slightly higher, but the difference was not significant.

## Introduction

Gene therapy is the process of insertion, alteration, and/or removal of targeted genes to and from an individual's cells to treat medical conditions. Typically, a copy of the coding zone of a target gene is carried by vectors to targeted tissues, organs, and cells. Genetic manipulation at the genome level (germ line gene therapy) is not considered ethical because of unpredictable effects, and genetic manipulation in somatic cells must be restrictively controlled to reduce the associated risks [1]. Local gene transfection to a limited region is one of the most convenient ways to enable such control.

The cochlea is traditionally considered as an ideal organ for local gene therapy because of its anatomical isolation and the fluid streaming internally [2]. At least theoretically, the isolation may limit the risk of side effects that may occur in other tissues and organs (*e.g.*, via the neoplastic process), and fluid movement may facilitate transportation of the targeted gene [3], [4], [5]. However, the effectiveness of this isolation has not been comprehensively evaluated in the situation of virus mediated cochlear gene transfection.

Genetic defects play a significant role in sensorineural hearing loss (SNHL), one of the most common neurological disorders [6]. Approximately 70% of genetic hearing loss disorders are non-syndromic hearing loss (NSHL), involving more than 100 identified genetic loci [7], [8]. In those cases, the deficit is selective to inner ears and may be corrected by local gene therapy in this isolated organ [9], [10].

Various vectors to transfer target genetic material into target tissue or cells have been investigated. At present, viral vectors are considered to have the best transfection efficiencies among vectors. Within this catalog, adenovirus (AV), adeno-associated virus (AAV), lentivirus, and herpes simplex virus have been investigated in the inner ears of mammals [11], [12], [13], [14]. Among these, AAVs are favored because they are not pathogenic or ototoxic, can transfect most cell types including neurons in the cochlea, and provide long-lasting expression of transfected genes. Moreover, different approaches for vector delivery have been evaluated. In a recent paper, we reported the use of a newly developed adeno-associated virus with mutations of surface-exposed tyrosine residues [15], in combination with digestion of the round window membrane (RWM) to achieve inner ear gene transfection in guinea pigs. The result was satisfactory and the method was minimally invasive [16].

Mouse models are extremely important and useful for the study of genetic hearing loss for several reasons, which have been extensively reviewed [17]. Briefly, their gene map is nearly complete, various genetic manipulations for hearing loss are available, and most mouse models faithfully mimic hearing impairments in humans [18], [19]. The technology used to generate genetically modified mice has been improved, and the methods are tractable and reproducible across laboratories [20], [21], [22], [23], [24]. Thus, it is reasonable that cochlear gene transfection conducted in laboratory mice should be the first step in research to correct genetic deficits in hearing loss.

In many cases, genetic hearing losses develop in the early stages of development and involve deformation of the auditory sensory organ [19]. This deformation cannot be corrected after it is established. Therefore, genetic interference to correct the deficit must be administered early (*i.e.*, before the onset of recessive nonsyndromic deafness) [25]. Early intervention presents a challenge in the mouse model, because the small size of the neonatal cochlea makes surgical manipulation extremely difficult. Potential rejection of the pup by the mother after surgery presents an additional problem. To our knowledge, only one study on cochlear gene transfection in neonatal mice has been reported [26]. In that brief report, cochlear transfection was conducted using AAV and AV. However, the cell transfection rate has not been quantitatively reported. The present study investigated cochlear gene transfection in neonatal C57BL/6J mice at day 7 after birth (P7). We compared the efficiency and safety of 2 AAV-mediated transfection methods: injection via RWM puncture, and trans-RWM diffusion after enzyme-induced digestion of the RWM.

## Materials and Methods

### Ethics Statement

C57BL/6J mice (Charles River Co. Canada), irrespective of gender but with body weights of 5–7 g were selected at postnatal day 7 (P7). This study was carried out in strict accordance with the recommendations set out in the Guide to the Care and Use of Experimental Animals, Canada. The protocol was approved by the Canadian Council on Animal Care (CCAC) at Dalhousie University, Halifax, Canada (Permit Number: 10-026). All surgery was performed under ketamine anesthesia, and all efforts were made to minimize suffering.

### Subjects and experimental protocol

We conducted a preliminary study to identify the highest concentration of collagenase that would dissolve the RWM without causing significant hearing loss, and to determine the best protocol to reduce mortality following surgery.

In the present study, we performed surgery [for RWM puncture or RWM partial digestion] on 38 pups at P7. Of those, 32 survived 2 weeks after surgery: 12 in the RWM-puncture group (of which 6 were used for surface preparation and 6 for the cross-sectional view), and 20 in the RWM partial digestion group (including 12 in the surface preparation condition and 8 in the cross-section view condition). Otological inspection was performed to rule out outer and middle ear abnormalities. Surgery for transfection of the marker gene (green fluorescent protein) was performed on 1 ear in each animal. The hearing status of each ear was evaluated 2 weeks after surgery using the ABR (auditory brainstem response) in a closed field. The animals were then sacrificed and the cochlea harvested for morphological examination, including surface preparation of the basilar membrane and saccule and frozen sections of the mid-modiolar area.

### Mut –AAV8 vector

The viral vector containing the green fluorescent protein (GFP) gene was generously provided by Dr. William Hauswirth of the Retina Gene Therapy Group, University of Florida. This virus contains a transgene cassette for GFP that is driven by the chicken *β*-actin (GBA) promoter. The recombinant AAV-8 (Y733F, titer: 4.5E+12) contains a mutation in surface-exposed capsid tyrosine residues [27]. This mutation largely reduces phosphorylation and subsequent ubiquitination of the AAV in the cytoplasm, and thus prevents proteasome-mediated degradation and improves intracellular traffic to the nucleus.

### Surgery

An aseptic protocol was followed for the surgery, which was carried out under anesthesia with ketamine (100 mg/kg) and xylazine (4 mg/kg; i.p.) (both from CDMV, Saint-Hyacinthe, QC, Canada). An arc incision was made in the postauricular area of the randomly selected ear. The other ear remained untouched. Blunt dissection was performed through the underlying soft tissue and auditory bulla, and the musculature was drawn to expose the posterior part of the auditory bulla. A hole was made on the bulla using microintraocular scissors with 90°-angled blades; this was enlarged using forceps until the round window niche was visible under the surgical microscope. In the trans-RWM group, 1 µL of collagenase I (C0130, Sigma, USA) solution (30 mg/mL) was dropped on the soft tissue that covered the round window niche and left there for 5 min before it was removed. Dental paper points were used to push the soft tissue away, revealing the underlying RWM. Then, 1 µL of the target concentration of collagenase solution (30, 60, or 90 mg/mL) was dropped into the RWM niche and left there for 10 min before it was replaced with 1 µL of the viral solution, which was applied on a piece of Gelfoam that covered RWM. Finally, the incision was sutured. The entire procedure took approximately 60 min. In the RWM-puncture group, glass needles with a tip diameter smaller than 10 µm (made using a micropipette puller) were used to puncture the RWM, and 1 µL of viral solution was then injected at the rate of 20 nL/sec using a microsyringe pump (Micro4; WPI, Kissimmee, USA).

### ABR tests

The animal was anesthetized with ketamine (80 mg/kg) and xylazine (4 mg/kg; i.p.) and kept warm using a thermostatic heating pad. The ABR test was performed in a closed field using Tucker Davis Technologies hardware and software (TDT, Alachua, FL, USA). The acoustic signal was generated digitally using an RP2.1 processor, amplified using an ES1 amplifier, and delivered via a broadband electrostatic earphone (EC1). The responses were picked up by subdermal electrodes; amplified ×20, and band-pass filtered between 100 Hz and 3 kHz. The signals were input to a real-time processor (RA16BA) and processed using BioSigRP software to an average 1,000 times in each trial. The threshold was evaluated at 8 and 32 kHz. At each frequency, stimuli were presented at 90 dB and then decreased in 5-dB steps until the threshold was approached, and then decreased in 5-dB steps until the ABR wave disappeared. The threshold was defined as the lowest intensity at which a visible and repeatable ABR wave was observed in 2 runs of averaging.

### Morphology

The pups were sacrificed with an overdose of pentobarbital (100 mg/kg; Animal Resources Centre, McGill University, Montreal, Canada). The temporal bones were harvested quickly and the bullae were opened. The cochleae were immersed in 4% paraformaldehyde in 0.1 M phosphate-buffered saline (PBS) for at least 4 h. The basilar membrane was dissected out, and then the stria vascularis, Reissner's membranes, and the membrana tectoria were removed. The saccule was dissected following removal of the otolith. The cross-sections were prepared by decalcifying the cochlea in 10% EDTA on a swing bed for 3 days at room temperature after fixation. The samples were then cryoprotected in graded sucrose in 0.1 M PBS (15%, 30%) overnight at 4°C and then embedded, frozen, and sectioned at a thickness of 6 µm.

For the immunohistochemical analysis of the surface preparation and frozen sections, the specimens were incubated in PBS–0.25% Triton X-100 for 40 min and then incubated overnight at 4°C with rabbit polyclonal GFP antibody (dilution 1∶100, NB600-308, Novus Biologicals, Littleton, CO, USA). The samples were rinsed the following day with PBS, incubated with Cy2-conjugated goat anti-rabbit IgG (dilution 1∶100, 111-225-045, Jackson ImmunoResearch, West Grove, PA, USA) for 60 min, followed by rinsing with PBS. Subsequently, the samples were double stained with TO-PRO-3 (dilution 1∶1000, T3605, Invitrogen, Carlsbad, CA, USA)or rhodamine phalloidin (dilution 1∶100, R415, Invitrogen). Transfected cochlear cells were counted under a Nikon light microscope with epifluorescence (Nikon ECLIPSE 80i, Tokyo, Japan), and images were captured with Zeiss LSM 510 META laser-scanning confocal microscope (Carl Zeiss, Jena, Germany).

## Results

### Survival rate

In a preliminary study, we investigated possible factors affecting the survival of 52 neonatal mice after surgery: removing the father from the colony (father isolation), sham surgery, and a combination of both. We found that mortality was largely the result of parental rejection following surgery, rather than the surgery itself. In the control group, in which surgery was performed on 9 of 13 pups: 1 died during the surgery, 8 (6 operated) were eaten by the parents in the 2-week period following surgery, and 4 survived (2 operated). In the father-isolation group, surgery was performed on 8 of 12 mice. Of the pups that underwent surgery: 1 died during surgery, 3 were eaten by the mother, and 4 survived 2 weeks after surgery. Of the 4 untreated pups, 3 were eaten by the mother and 1 survived 2 weeks. In the sham surgery group, the RWM was exposed in 9 of 13 pups, and a skin incision alone was made in the remaining 4. The survival rate of this group was better than that of the control group: 7 of 13 pups survived 2 weeks after the operation and 5 of the survivors were in the RWM exposure group. Survival rates improved when father isolation and sham surgery were combined: 11 of 14 pups survived 2 weeks, including 7 of the 10 that received surgery (Table 1).

**Table 1:** Survival after surgery by experimental group.

Group	Within group allocation				Number of mice surviving 2 weeks post- surgery			
	Total	Op	Non-op	S-op	Total	Op	Non-op	S-op
Control	13	9	4	N/A	4	2 (22%*)	2	N/A
Father isolation	12	8	4	N/A	5	4 (50%*)	1	N/A
Sham surgery team	13	9	N/A	4	7	5 (56%*)	N/A	2
Both	14	10	N/A	4	11	7 (70%*)	N/A	4

* Survival % for animals that received the surgery. Op: operated, S-op: sham operation

### RWM treatment and hearing function

**Figure 1:**
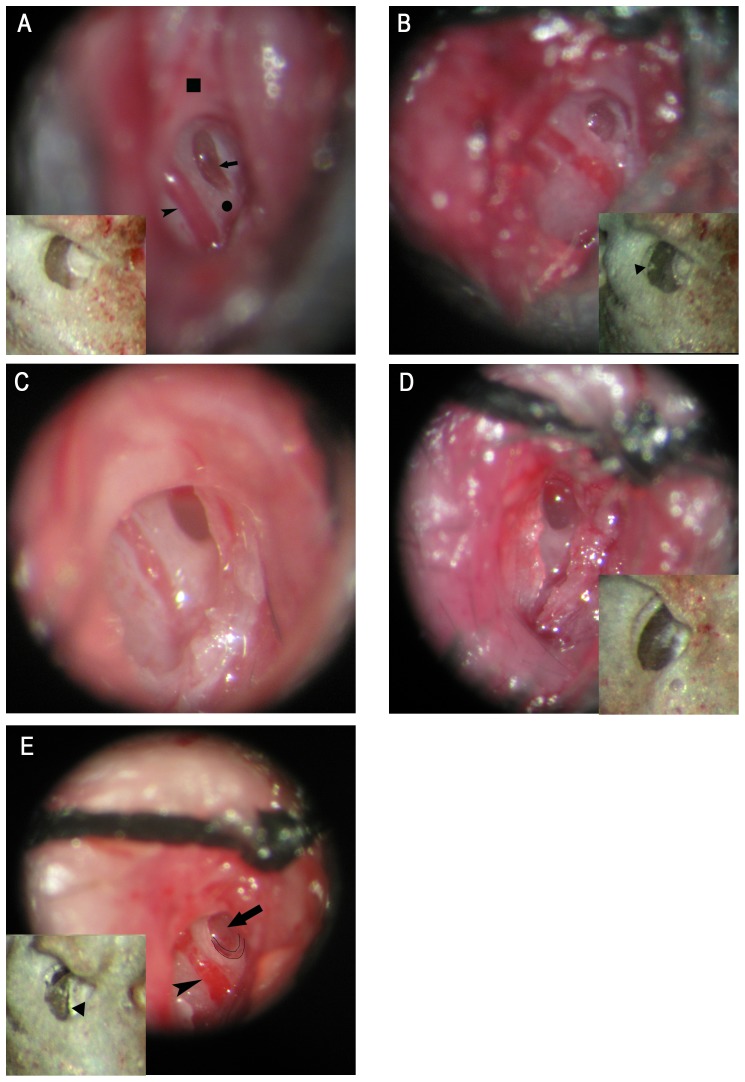
Views of surgical areas showing the conditions of RWM after different treatments. The insert of each image performed RWM in vitro correspondingly. A: control RWM freshly exposed. The RWM was partially covered by a piece of bone, which was routinely removed during surgery. B: Punctured RWM. The punctured point was covered by a small amount of fluid. C: RWM treated with 30 mg/mL collagenase I, revealing intact RWM with slight congestion. D: RWM treated with 60 mg/mL collagenase I. RWM was intact, but apparent congestion was observed in the RWM and nearby soft tissues, and a scar was seen in the middle of RWM in vitro. E: RWM treated with 90 mg/mL collagenase I, resulting in a large perforation of the RWM, indicated by the crosses. Arrow, RWM; arrowhead, stapedial artery; square, wall of the bulla; circle, lower edge of the round window niche; triangle, the perforation observed in RWM. RWM: round window membrane.

The impact of RWM treatment on hearing function was evaluated in 29 neonatal mice. After treatment with collagenase I for 10 min, the reagent was removed, and the conformation of RWM was observed under a surgical microscope. No RWM damage was evident after treatment with 30 or 60 mg/mL of the enzyme solution, with the exception of slight congestion in RWM and the surrounding soft tissues. However, a large RWM perforation was observed after treatment with 90 mg/mL. In the RWM-puncture group, no macroscopic perforation was observed after puncture with a glass needle. After the needle was removed, the puncture point was covered by a small amount of fluid (most likely perilymph leaked from the cochlea), and the RWM was slightly sunken compared with that of the control (Fig. 1.). However, we did not observe a continuous leak of the perilymph.

**Figure 2:**
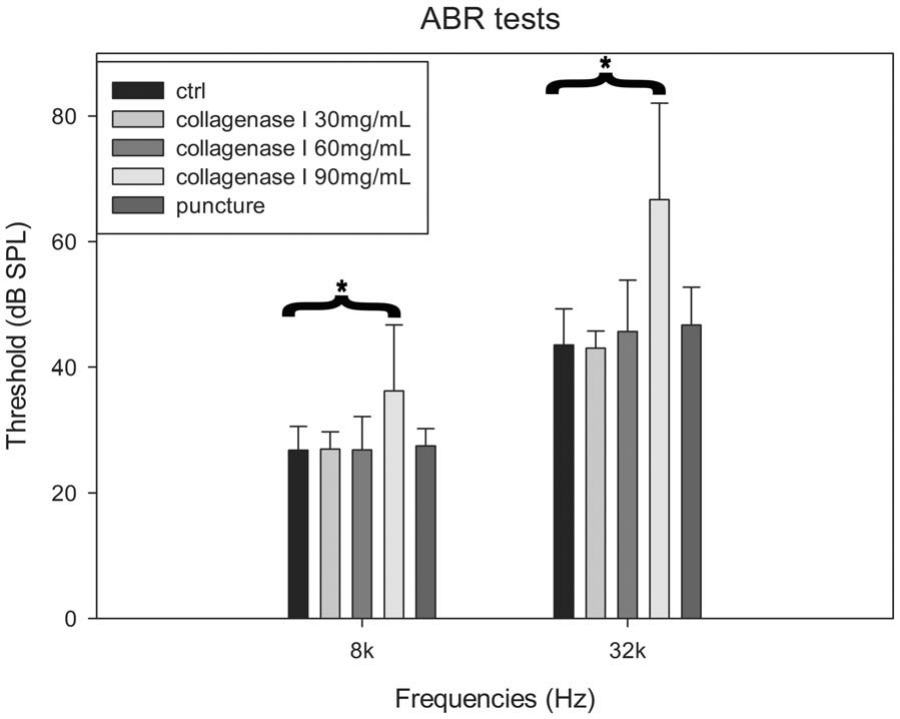
Comparison of auditory brainstem response (ABR) threshold across groups. *: a significant difference (*p*<0.01) between the control and the ears treated with 90 mg/mL collagenase.

To evaluate the effect of RWM digestion on hearing, ABR (8 and 32 kHz) was evaluated in 25 of 29 mice who survived 2 weeks postsurgery (Fig. 2). This analysis revealed no significant threshold differences between the operated and contralateral ears (not operated) in either the RWM-puncture group or the groups that received collagenase I at 30 and 60 mg/mL (two-way repeated-measures analysis of variance or ANOVA; *p*>0.05). In contrast, the threshold for ears treated with collagenase I at 90 mg/mL was significantly higher than that of the control ears; 12.1±4.4 (mean±SE) dB at 8 kHz and 26.7±6.8 dB at 32 kHz (*p*<0.01, two-way repeated-measure ANOVA).

### Inner ear transfections

In total, 38 mice were recruited for the formal transfection experiment and 32 survived 2 weeks after the virus injection. Of these, 18 were used to assess AAV transfection in the basilar membrane using the surface preparation, and the remaining 14 were used to verify the distribution of AAV transfection in the cross-sectional views of the cochlea.

**Figure 3:**
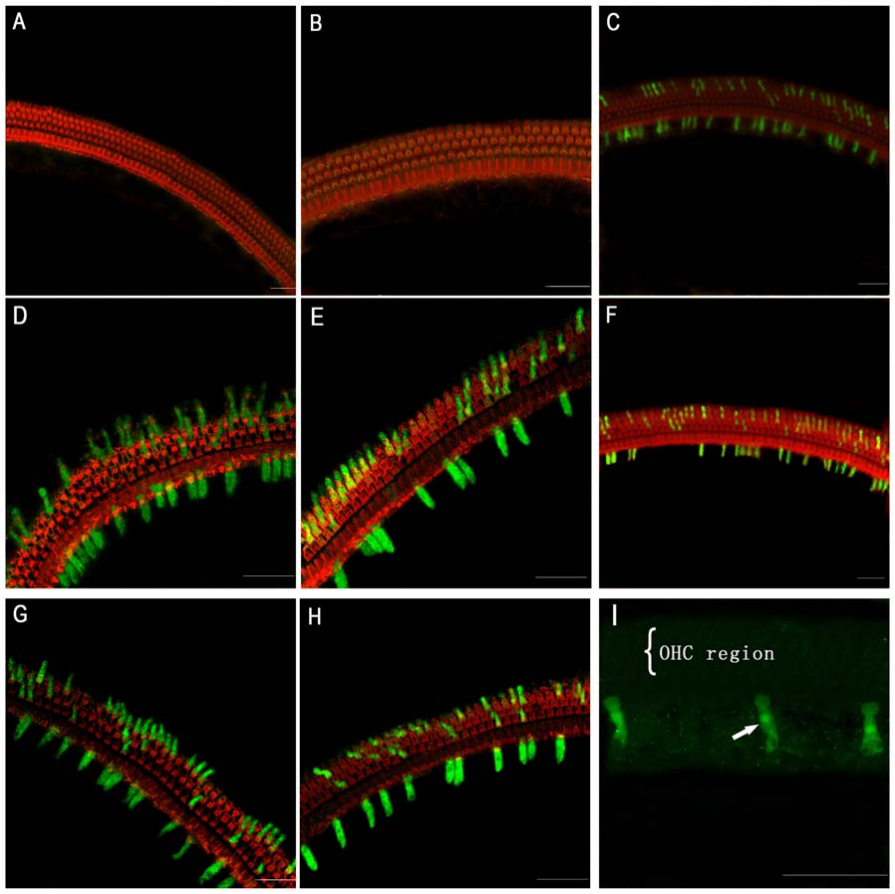
Mut-AAV-GFP transfection in the hair cells of neonatal mice 14 days postinoculation. A and B: Negative transfection in the hair cells of the collagenase 30 mg/mL group. C–E: Transfection in hair cells at different magnitudes in the collagenase 60 mg/mL subgroup, D: Apical turn, E: Basal turn. F–H: Transfection in hair cells viewed at different magnitudes in the RWM-puncture group. G: Apical turn, H: Basal turn. I: Sparse IHC transfection (arrow) seen in an ear contralateral to that receiving virus via RWM puncture. Scale bars = 50 µm. RWM: round window membrane.

**Figure 4:**
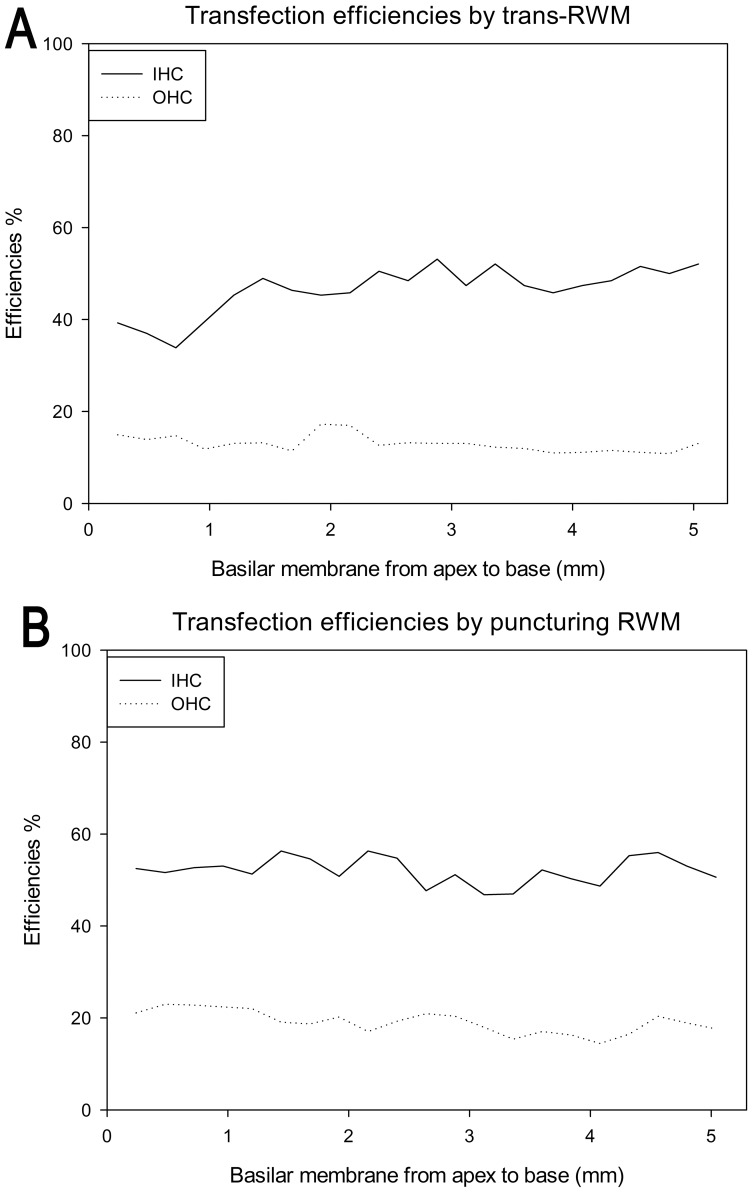
Cochleograms showing transfection efficiency in the OHCs and IHCs A: trans-RWM group, B: RWM-puncture group. IHCs: inner hair cells; OHCs: outer hair cells; RWM: round window membrane.

In the surface preparation, green fluorescent protein (GFP) staining was evident in the cochlear cells of the RWM-puncture group (*n* = 6; Figs. 3C–E) and trans-RWM 60 mg/mL collagenase I subgroup (*n* = 8; Figs. 3F–H), but not in the 30 mg/mL collagenase I subgroup (*n* = 4; Figs. 3A–B). Digestion with enzyme at 90 mg/ml was not employed because the preliminary experiment revealed that it caused RWM perforation. In the 60 mg/mL trans-RWM subgroup, both the inner hair cells (IHCs) and outer hair cells (OHCs) were partially transfected in the whole basilar membrane (IHCs, 47±7%; OHCs, 17±4%) and in the basal turn (IHCs, 49%; OHCs, 13%), suggesting a similar longitudinal transfection (Figs. 3F–H, Fig. 4A). In the RWM-puncture group, the corresponding transfection rates were 58±4% and 19±4% for IHCs and OHCs, respectively, across the entire cochlea and 51% and 18% for the basal turn (Figs. 3C–E, Fig. 4B). Sparse IHC transfection was observed in the contralateral ears of 2 mice in this group (Fig. 3I). No transfection signals were captured in the saccule of either the trans-RWM or RWM-puncture group (Figs. S1A–B).

**Figure 5:**
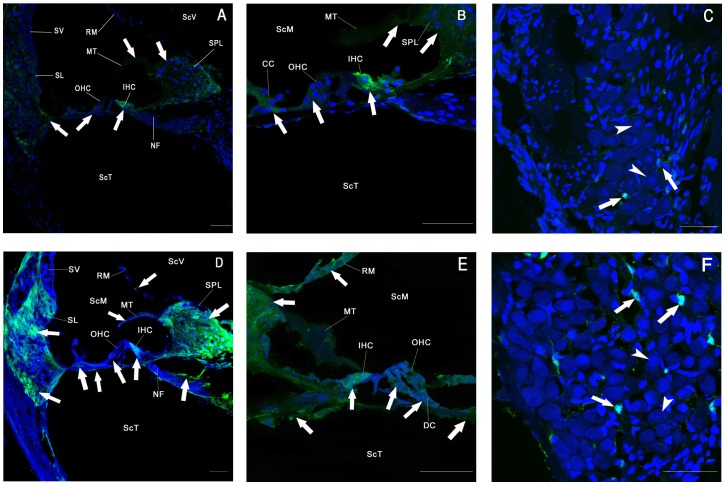
Cross-sectional view of the basal turn of cochlea showing cochlear transfection. In the trans-RWM subgroup of RWM treatment with 60 mg/mL collagenase. A–C: Strong GFP signals were observed in the IHCs, Claudius cells, membrana tectoria, spiral limbus, and spiral ligament; GFP staining was weak in the OHC (A and B). Few GFP-positive type II SGNs were found (arrows in C), and no staining was observed in type I SGNs. RWM-puncture group D–F: the relative transfection across different cell types and locations was similar to the trans-RWM group, but the GFP signal was generally stronger in the RWM-puncture group. GFP-positive type II SGNs (arrows in F). Arrowhead: type I SGNs. ScV: scala vestibule; ScT: scala tympani; ScM: scala media; OHC: outer hair cell; IHC: inner hair cell; SGNs: spiral ganglion neurons; CC: Claudius cell; DC: Deiters cell; SL: spiral ligament; SV: stria vascularis; MT: membrana tectoria; RM: Reissner's membrane; SPL: spiral limbus; NF: nerve fibers. Scale bars = 50 µm.

The cross-sectional images revealed a wide distribution of GFP transfection in various cell types and locations, including OHCs, IHCs, supporting cells, the membrana tectoria, and spiral limbus in the ears of the trans-RWM 60 mg/mL collagenase I subgroup (*n* = 8; Figs. 5A–C) and in the RWM-puncture group (*n* = 6; Figs. 5D–F). The transfection rate in the spiral ganglion neurons (SGNs) in Rosenthal's canal was slightly higher in the RWM-puncture group (Fig. 5F) than in the trans-RWM subgroup (Fig. 5C) The SGN transfection rate, however, was low in both groups.

## Discussion

The present study investigated the efficiency of 2 methods of gene transfection in neonatal mice. The investigation of gene therapy in mice is justified by the importance of mouse models in the research of genetic hearing loss and the early onset of this loss in many cases. After examining the challenges of high mortality after transfection surgery and the difficulty of operating on the small cochleae of neonatal mice, this study compared the transfection efficiency of 2 gene transfection approaches: RWM puncture; and trans-RWM after digestion. Using appropriate procedures and a new generation of AAV vector (with mutations of surface-exposed tyrosine residuals), we were able to obtain satisfactory transfection of the marker gene to the IHCs and OHCs, the most important cell types in the cochlea. However, transfection to type I SGNs was surprisingly low compared with that previously reported in adult guinea pigs [16]. Moreover, we found the trans-RWM approach to be slightly less effective, and the outcomes were more varied.

Cochlear gene transfection using AAV vectors is relatively easy and safe. In a previous study, we found the trans-RWM approach to be effective and minimally invasive in guinea pigs [16]. However, successful cochlear gene transfection in neonatal mice had the additional problem of a high postoperative mortality rate, primarily as a result of infanticide by the parents. The reasons for this are not clear, as no comprehensive study of this behavior is available. However, as it varies across strains, infanticide is likely dependent on some genotypic features of the animals [28], [29]. When males remain in the colony, the colony is more aggressive, and weaker pups are more likely to be eaten by the parents. In our preliminary study, we tested the effect of 2 factors and their combination: removing the father and sham surgery. The purpose of the sham surgery was to equalize the overall health status of all pups in the colony. Although this was not intended to be a systematic study, the preliminary study demonstrated that a combination of the 2 procedures significantly improved the survival rate of the neonates. Therefore, father isolation and sham surgery were used in the colonies involved in the gene transfection experiments.

Several procedures were considered beneficial and were adopted in this study. First, the mice were deeply anesthetized because of the separation of sternocleidomastoideus [30]. Our aesthetic dose was adequate to maintain a deep sleep for approximately 40–60 min. Second, we did not use gas inhalation for anesthesia because the muzzle required for gas may have interfered with the operation [31]. A further difficulty in the surgery was the exposure of the RWM, because it was largely covered by soft tissue. The narrow operating field made it difficult to separate the soft tissue mechanically without damaging the underlying RWM. Digestion using collagenase appears to be an effective way to overcome this problem. After digestion, the soft tissue became spongy and could be dissected with a paper tip.

Unlike microinjection through the bony wall of the cochlea into the scala media, the local transfection used in the present study did not cause cochlear damage [26], [32]. We found no threshold differences between the operated and control ears during the ABR test in either the trans-RWM or RWM-puncture groups. Application of collagenase solution facilitated dissection of the soft tissue that covered the RWM, and it partially damaged the RWM, which temporarily increased membrane permeability to the viral vector. The high regenerative potency of epithelial cells and connective tissues ensured that the RWM damage induced by collagenase digestion soon healed, as demonstrated in our previous report [16]. This autonomic healing explains why the hearing function remained unchanged. Moreover, the ABR test results, which revealed no threshold difference between operated and contralateral ears, indicated that the injury caused by RWM puncture had healed by 2 weeks post-surgery. We used a micromanipulator to hold and advance the glass needle so that the RWM perforation was extremely small, as determined by the needle tip diameter, which was approximately 10 µm. We determined that this hole was well sealed after the needle was removed because any observed perilymph leakage was not significant. RWM treatment using 90 mg/mL collagenase caused over-digestion and significant hearing loss.

A comparison of the trans-RWM and RWM-puncture methods in terms of hearing function maintenance, transfection efficiency, and surgical difficulty revealed that both procedures preserved hearing function equally well, although the trans-RWM method did not disturb the perilymph with the administration of a large volume of exogenous agent. Our findings suggest that transfection via RWM puncture is more effective than that via the trans-RWM method, based on equal amounts of viral solution. However, more viral solution reached the perilymph in the RWM puncture group than in the trans-RWM diffusion group. The difficulty of the surgery was comparable in both approaches. The collagenase treatment and application of the virus increased the surgical time in the trans-RWM procedure. However, the vertical insertion of the needle tip across the RWM, which is hidden approximately 1 mm below the RW niche, required great caution and skill.

Our results indicated that cochlear transfection in neonatal mice is characterized by an even longitudinal transfection in the hair cells and low transfection efficiency in the SGNs. The mechanisms underlying the even transfection are not clear. In guinea pigs, transfection rates are reportedly higher in the basal turn, which is closer to the site of virus application [12]. One explanation for this is that the vector was not fully diffused to the apex of the cochlea. However, evidence for quick longitude transportation of material in the perilymph has been reported, so the diffusion distance does not explain the transfection gradient [33]. AAV entry into the cell is mediated by special receptors; differences in receptor distribution may establish a gradient of cellular tropism to AAVs along the cochlea [34]. The third possible reason is a difference in basilar membrane permeability to AAVs across the cochlea. However, no evidence for AAV tropism or basilar membrane permeability along the cochlea has been reported in either guinea pigs or mice.

The reasons for the low SGN transfection rate in neonatal mice observed in the present study are also not clear. 2 pathways have been proposed for AAV transportation to the organ of Corti: (1) diffusion across the basilar membrane, which lacks tight junctions [35], [36], [37]; and (2) transport along nerve fibers via the habenula perforata after AAV transfection of the SGNs. The 2nd approach was proposed to explain the higher transduction rates in IHCs than in OHCs following AAV transfection, suggesting that IHCs receive richer innervations from thick type I fibers. However, no clear evidence exists to support this postulation. The low SGN transfection rate observed in the present study and in others [12] essentially refutes the possibility of the latter pathway.

Some untreated ears in the RWM-puncture group exhibited sparse IHC transfection. This was likely the result of the connection between the cochlear fluid and cerebrospinal fluid (CSF) via the cochlear aqueduct [38], and the fact that the 1 µL of viral solution injected in the present study was greater than the perilymph volume, which is reportedly 0.62 µL in adult mice [39]. We injected a large volume of viral solution to maximize gene transfection. However, the original perilymph was completely replaced by the viral solution, and some may have leaked into the CSF. Leakage of the virus into the CSF suggests that the cochlea, at least in neonatal mice, is not entirely isolated and local gene transfection into the cochlea may have unpredictable effects in the central nervous system. Thus, the quantity of viral solution should be carefully planed to balance transfection and the risk of an unplanned leakage into the CSF. An advantage of the trans-RWM method is that leakage into the CSF is not a potential problem.

In summary, a high survival rate was obtained when father isolation and sham surgery were combined. The present study demonstrated satisfactory gene transfection to hair cells in neonatal mice at P7 via 2 methods, without causing significant hearing loss. The RWM puncture did not have an adverse effect on hearing preservation, and the ABR test results of this group were similar to those of the trans-RWM group. This was most likely the result of autonomic healing in the RWM of young mice. The outcome of our study paves the way for cochlear gene therapy in neonatal mice using AAV vectors.

## Supporting Information

Figure S1 **Comparison of AAV-mediated inner ear transfection in neonatal mice and adult guinea pigs.** Neither the trans-RWM nor the RWM-puncture approach achieved transfection in the saccule of neonatal mice (A and B respectively). In adult guinea pigs, satisfactory hair cell transfection was achieved in the saccule (C) and organ of Corti (D) via the cochleostomy approach. Scale bars = 50 µm. Transfected cells were not observed in the saccule of neonatal mice in either the trans-RWM or RWM-puncture group (Figs. S1A–B). However, in previous research involving adult guinea pigs, we found high transfection efficiency in the hair cells of the saccule (Fig. S1C) and the organ of Corti (Fig. S1D), using the same viral vector (mut-AAV8) but delivered via cochleostomy.
(TIF)
