# Supplementary figures and images for: Inner Ear Gene Transfection in Neonatal Mice Using Adeno-Associated Viral Vector: A Comparison of Two Approaches
Source: PLoS One. 2012 Aug 17;7(8):e43218. doi: 10.1371/journal.pone.0043218 (PMC3422324; doi:10.1371/journal.pone.0043218)

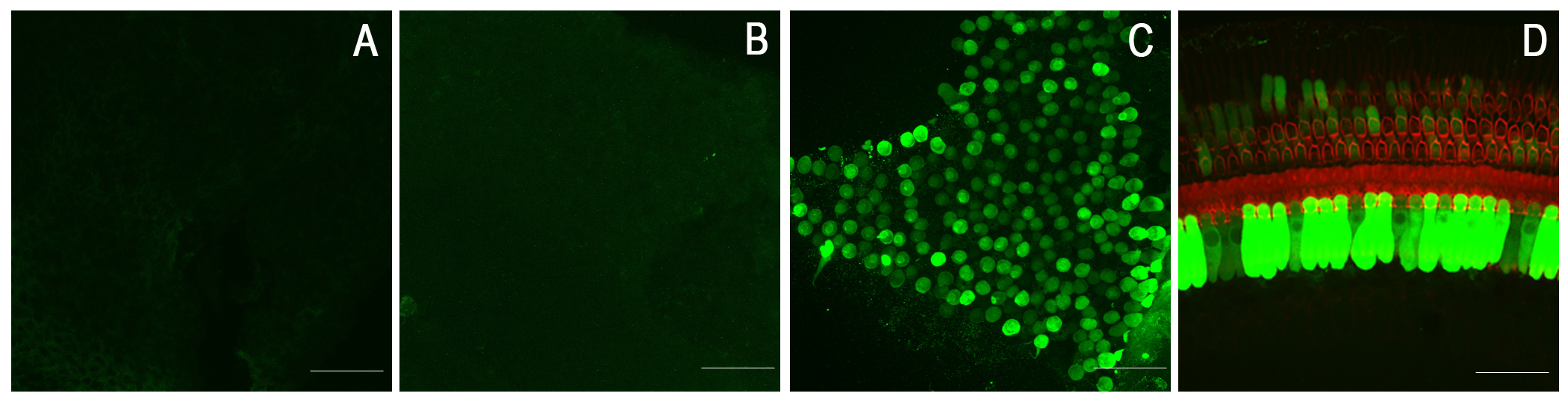

Supplement: Figure S1 — Comparison of AAV-mediated inner ear transfection in neonatal mice and adult guinea pigs. Neither the trans-RWM nor the RWM-puncture approach achieved transfection in the saccule of neonatal mice (A and B respectively). In adult guinea pigs, satisfactory hair cell transfection was achieved in the saccule (C) and organ of Corti (D) via the cochleostomy approach. Scale bars = 50 µm. Transfected cells were not observed in the saccule of neonatal mice in either the trans-RWM or RWM-puncture group (Figs. S1A–B). However, in previous research involving adult guinea pigs, we found high transfection efficiency in the hair cells of the saccule (Fig. S1C) and the organ of Corti (Fig. S1D), using the same viral vector (mut-AAV8) but delivered via cochleostomy. (TIF) [file pone.0043218.s001.tif]
